# Dynamic Properties of β-Casein Fibril Adsorption Layers at the Air–Water Interface

**DOI:** 10.3390/polym17081075

**Published:** 2025-04-16

**Authors:** Anastasiya R. Rafikova, Olga Y. Milyaeva, Giuseppe Loglio, Reinhard Miller, Zhili Wan, Boris A. Noskov

**Affiliations:** 1 Department of Colloid Chemistry, St. Petersburg State University, Universitetsky pr. 26, 198504 Saint Petersburg, Russia; nastya.rafikova.2000@mail.ru (A.R.R.); b.noskov@spbu.ru (B.A.N.); 2 Institute of Condensed Matter Chemistry and Technologies for Energy, 16149 Genoa, Italy; giuseppe.loglio@ge.icmate.cnr.it; 3 Institute of Condensed Matter Physics, Technische Universität Darmstadt, D-64289 Darmstadt, Germany; reinhard.miller@pkm.tu-darmstadt.de; 4 School of Food Science and Engineering, South China University of Technology, Guangzhou 510640, China; zhiliwan@scut.edu.cn

**Keywords:** β-casein fibrils, protein aggregation, air–water interface, adsorption layers, dilational surface visco-elasticity, ellipsometry, AFM

## Abstract

Although the formation of the layers of fibrillar aggregates at liquid–liquid and liquid–gas interfaces can significantly increase the stability of disperse systems, like foams and emulsions, any information on their structure and properties is rather limited. In the present work, surface properties of the adsorption layers of fibrils of intrinsically disordered β-casein are investigated. For unpurified dispersions of the fibrils of this protein, the dynamic surface elasticity proved to be close to the values for the native protein solutions. This behavior is typical for dispersions of fibrils of globular proteins. However, previously studied fibrils of another intrinsically disordered protein, κ-casein, do not demonstrate this similarity. The contribution of β-casein fibrils to the dynamic surface properties becomes noticeable only after the purification of the dispersions from impurities of high surface activity. The dynamic surface elasticity increases up to 48 mN/m after two purification cycles, i.e., to values 4 times higher than the steady-state values of native protein adsorption layers at the same protein bulk concentrations.

## 1. Introduction

Studies of amyloid fibrils over the past century has been mainly focused on their role in pathological processes in the human body [1,2]. At the same time, the discovery of non-pathological, functional amyloids and their unique properties has opened up opportunities for their use in addressing various practical challenges. This has inspired researchers to develop artificial materials based on the fibrils for the use in biotechnology, medicine, and the food industry [3,4,5,6,7,8,9,10].

Milk contains amyloidogenic casein proteins, and thereby can be used to create various novel products with protein fibrils [11,12]. The primary protein in milk is casein. It is composed of four distinct casein types: α_s1_-, α_s2_-, β-, and κ-caseins. These caseins are present in a ratio of approximately 4:1:3.5:1, respectively [13,14].

Caseins, unlike other milk proteins, can bind amorphous calcium phosphate and insignificantly increase its solubility [15]. They are also prone to aggregation because of their amphiphilic nature and partial separation of the hydrophilic/charged and hydrophobic groups in their molecules [16,17].

α_s2_- and κ-caseins readily aggregate into amyloid fibrils in vitro under physiological conditions [16,17,18]. At the same time, milk typically does not contain amyloids, since β- and α_s1_-caseins act as highly effective molecular chaperones [19,20,21,22,23]. Instead of forming amyloid fibrils, a large number of casein molecules and amorphous calcium phosphate nanoclusters form casein micelles [15,24,25].

Although β-casein is recognized for its ability to act as a molecular chaperone, it can also contribute to the formation of fibrils [18,26,27,28,29]. Pan et al. showed that β-casein can assemble into amyloid fibrils under acidic conditions and high temperatures in vitro [18]. The presence of heparin sulfate and metal ions, such as Zn^2+^ and Ca^2+^, can facilitate this process [28,29].

The dimensions of β-casein fibrils are typical for these aggregates [26]. X-ray diffraction results show a pair of diffraction patterns corresponding to the distance between hydrogen bonding in adjacent β-strands perpendicular to the fibril axis (meridional reflections at ~4.7 Å) and the distance between β-sheets parallel to the fibril axis (equatorial reflections at ~10 Å). The core structure of protofilaments is formed by face-to-face stacked β-sheets. The viscosity of β-casein fibril dispersions is 10 times higher than the viscosity of κ-casein dispersions, and 5 times higher than the corresponding values for β-lactoglobulin dispersions [18], indicating the increased ability for gel formation in the former systems.

The stability of disperse systems is connected to a significant extent with the dynamic properties of liquid–fluid interfaces [30]. The impurities of unreacted proteins and polypeptides pose high surface activity and strongly influence the surface properties of fibril dispersions [31,32]. The surface properties of fibril dispersions without purification are similar to those of native protein solutions [33,34]. At the same time, the adsorption layers of purified lysozyme fibrils at the liquid–gas interface exhibit a dynamic surface elasticity of approximately 115 mN/m, which is about 40% higher than that of the native protein adsorption layers (80 mN/m) [34]. Information on the dynamic surface properties of the fibril dispersions of intrinsically disordered proteins compared to systems containing fibrils of globular proteins is quite limited [35]. One can expect that they will differ from the characteristics of native protein solutions, as well as from the characteristics of dispersions of globular protein fibrils.

This study focuses on the dynamic surface properties of aqueous solutions containing β-casein fibrils and the mechanism of formation of adsorption layers in these systems. Our findings can contribute not only to an improved understanding of adsorption layer properties but also to the development of novel functional materials based on disordered proteins.

## 2. Materials and Methods

### 2.1. Materials

β-casein (*M_w_* ≈ 24,000 Da) and Thioflavin T (ThT) (both from Sigma-Aldrich, Germany) were used without further purification. Stock solutions with a concentration of 0.5 mg/mL were used to prepare solutions of native protein by dilution with phosphate buffer. Stock solutions were stored in the refrigerator at 4 °C for a maximum of seven days.

Phosphate buffer solutions with pH 7.0 (I = 0.02 M) were prepared using NaH_2_PO_4_ and Na_2_HPO_4_ (Sigma-Aldrich, Taufkirchen, Germany) in triply distilled water. For experiments with varying ionic strength, NaCl (Vekton, Saint Petersburg, Russia) was added to the solutions under study. In order to remove organic impurities, NaCl was calcined at 750 °C.

### 2.2. Sample Preparation

The fibril preparation was described previously [18]. β-casein was dissolved in HCl aqueous solution with pH 2 to obtain a protein concentration of 1 wt. %. The solution was incubated in a thermostat at 90 °C with stirring (300 rpm) for 48 h. The resulting turbid light dispersion was rapidly cooled in a water–ice mixture to interrupt the reaction. The concentration of the dispersion was determined by drying a certain volume at 60 °C for 2 days and weighing the remaining dry material.

After synthesis, besides β-casein fibrils, the obtained dispersions contain unreacted protein and hydrolyzed peptides. The details of the dispersion composition and concentrations of the impurities are presented in Table S1, Figure S1 and Figure S2 in the Supplementary Information. Fibril dispersions were purified by centrifugation at 10,000× *g* for 40 min. After centrifugation, the supernatant was replaced by triple distilled water and fibrils were resuspended by shaking. To obtain a higher degrees of purification, this procedure was repeated once and twice, respectively.

### 2.3. Thioflavin T Fluorescence Assay of Fibril Formation

The ThT fluorescence assay was applied to confirm the fibril formation and to study the kinetics of this process [36,37]. Eight samples of β-casein solutions at different incubation times (0, 0.75, 1.5, 3.5, 18, 24, 42 and 48 h) were diluted by phosphate buffer solutions (pH 7.0, I = 0.02 M) to a total protein concentration of 2 × 10^−5^ M. Then, ThT was added to the samples under investigation to the final concentration of 2 × 10^−5^ M.

A HORIBA spectro-fluorimeter (Japan) was used to obtain the fluorescence spectra of ThT. The spectra were recorded in the range of 465–650 nm with an excitation wavelength of 448 nm. The fluorescence intensity (F) of ThT was normalized according to its maximum value at 480 nm for the sample incubated for 24 h (F_24_), considering the signal for the native β-casein solution (F_0_), as follows:

(1) $$f_{t}=\frac{F-F_{0}}{F_{24}-F_{0}}$$

The induction period t_lag_ and the time to reach half of the maximum fluorescence intensity t_1/2_ were calculated using the approach described in [18,38].

### 2.4. Dynamic Surface Elasticity and Dynamic Surface Tension

The dilational dynamic surface elasticity was measured using the oscillating barrier method. An ISR instrument (KSV NIMA in Espoo, Finland), equipped with a Wilhelmy plate, allows recording of the surface tension oscillations induced by periodical compressions and expansions of the surface area with an accuracy of ±0.2 mN/m. The surface area changes are produced by motion of the Teflon barriers along the Langmuir trough. If surface tension oscillations can be described by a harmonic law, the dynamic surface elasticity ε is defined as the ratio of the amplitudes of surface tension oscillations ∆γ and the relative surface area $∆A/A_{0}$:

(2) $$\varepsilon =\frac{∆\gamma }{∆A/A_{0}}$$

Since, in general, *ε* is a complex quantity, its real *ε_r_* and imaginary *ε_im_* parts can be connected with the phase shift *φ* between the oscillations of the surface tension and surface area [39].

For the systems under study, ε_r_ was much greater than ε_im_. Thus, only the results for ε_r_ are discussed below. The oscillation frequency and area amplitude of the Teflon barriers equaled 50 mHz and 5%, respectively.

### 2.5. Atomic Force Microscopy

The microstructure of the adsorption layers was characterized by atomic force microscopy (AFM). The images were obtained using a NTEGRA Spectra NT–MDT atomic force microscope (Moscow, Russia). The measurements were performed in the tapping mode. To prepare samples, adsorption layers were transferred from the water surface to a freshly cleaved mica surface by the Langmuir–Schaeffer method, and then left to dry in a desiccator for three days.

### 2.6. Brewster Angle Microscopy

Brewster angle microscopy (BAM) was used to evaluate the morphology of the surface layers at a mesoscopic level. All measurements were performed using a BAM1 instrument (Nanofilm Technology, Göttingen, Germany) equipped with a 10 mV He-Ne laser.

### 2.7. ζ-Potential Measurements

A Zetasizer ZS Nanoanalyzer (Malvern, Worcestershire, UK) was used to obtain information on the ζ-potential of the particles in the bulk. The scattering angle during all measurements was kept constant at 173°.

### 2.8. Statistical Analysis

All experiments were conducted in triplicate to ensure the reliability and reproducibility of the results. Standard deviations are represented as percentage values in the figure captions.

## 3. Results and Discussion

**Figure 1:**
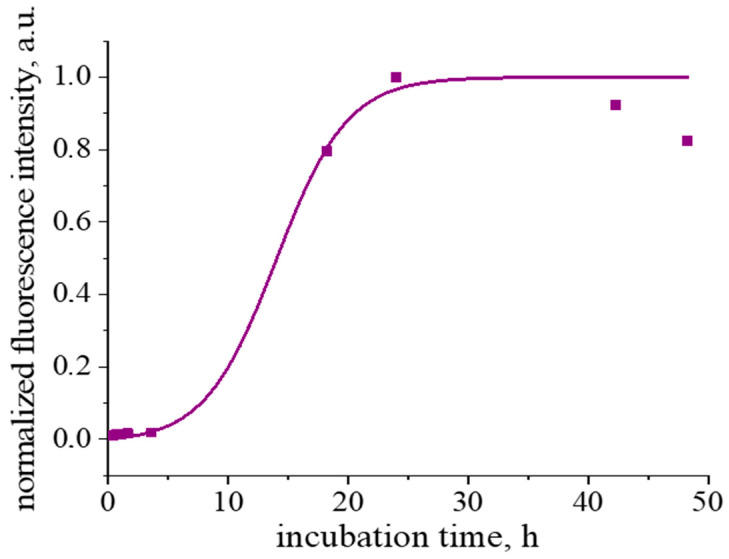
Kinetic dependence of normalized ThT fluorescence intensity. The purple line corresponds to the fitting curve.

The fibril formation at pH 2 and a temperature of 90 °C is accompanied by a noticeable induction period (Figure 1 and Figure S3A). Similar behavior has been observed previously for fibril formation at pH 6–7 and a temperature of 65 °C [26]. In this case, t_lag_ is approximately 8 h and t_1/2_ approaches 4 h.

A strong increase in ThT fluorescence at 480 nm is observed after heating of β-casein solutions for 24 h (Figure 1 and Figure S3A), when the maximum in the excitation spectrum shifts from 412 nm, which is typical for unbound ThT molecules [36,40], to 448 nm (Figure S3B). Such spectral changes are caused by the ThT interactions with an extended β-sheet structure [36,40], confirming the fibril formation. Note that, when a β-casein solution is heated for more than 24 h, the fluorescence intensity does not reach a plateau, which is typical for a fibril formation [41], and starts to decrease (Figure 1). Similar behavior was also observe for fibrils of some other proteins [38,42,43]. This peculiarity can be connected with a slow gel formation [38] or some destruction of the β-sheet structure due to hydrolysis [42]. Besides, few fibrils can form larger elongated aggregates, thereby reducing the available surface for ThT binding [44].

**Figure 2:**
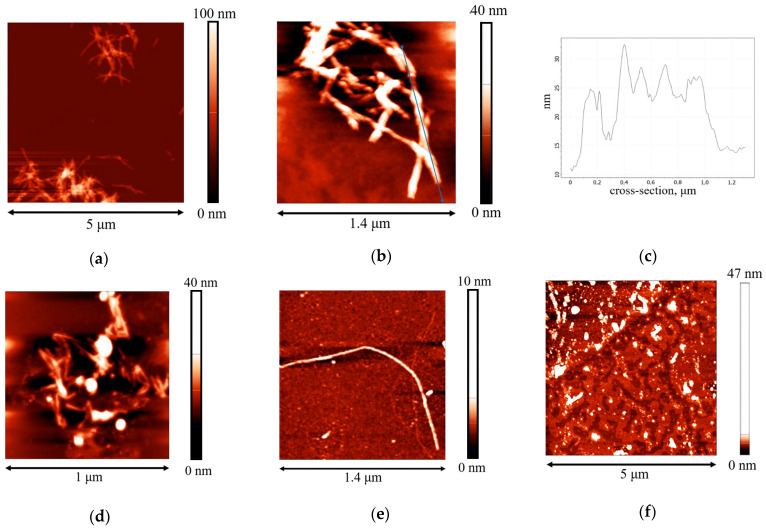
AFM images of (**a**,**b**) β-casein fibrils after preparation at pH 2 and a temperature of 90 °C for 48 h; (**c**) cross-section corresponding to the blue line; (**d**,**e**) adsorption layers of two-times purified dispersions with a concentration of 0.01 mg/mL at pH 7.0 with 0.1 M NaCl; (**f**) adsorption layer of twice purified dispersion with a concentration of 0.01 mg/mL at pH 7.0 with 0.1 M NaCl after compression by 80% of the initial surface area.

The length of the obtained β-casein fibrils does not exceed 1.2 μm (Figure 2a,b). One can observe also some periodic humps along the fibrils with a distance of approximately 200 nm between them (Figure 2c). Such periodic local thickenings have been observed earlier for some other protein fibrils and can be caused by the twisting of a few protofilaments at some points along the main axis of the aggregate [45].

In general, the aggregates of β-casein are much shorter than κ-casein fibrils [35], in agreement with the weaker β-casein ability in forming fibrils. This feature can be a consequence of a large number of proline residues in the hydrophobic C-terminus [16,46]. The core of β-casein fibrils contains hydrophobic peptides [26], like the cores of some other fibrils [47]. The proline residues limit the chain flexibility and complicate the formation of α-helices and β-sheets, and therefore the fibril formation [26].

The unpurified fibril dispersions contain, in addition to aggregates, a significant number of hydrolyzed peptides and unreacted protein molecules. These impurities influence significantly the dynamic surface properties of the dispersion [34].

**Figure 3:**
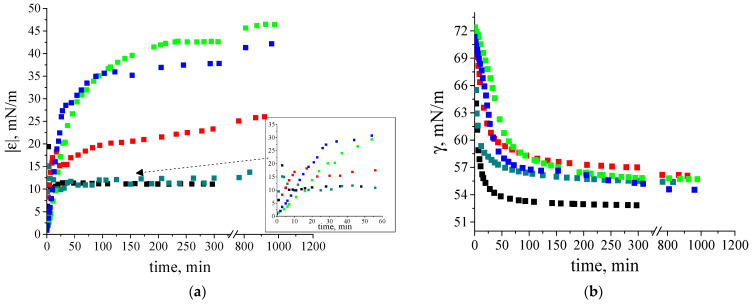
Kinetic dependence of (**a**) dynamic surface elasticity and (**b**) dynamic surface tension of native β-casein solution (black squares), unpurified fibril dispersion (dark cyan squares), one-time (red squares), two-times (green squares) and three-times (blue squares) purified fibril dispersions with a concentration of 0.01 mg/mL at pH 7.0 and with 0.1 M NaCl. The standard deviation of the measured parameters was lower than 5%. The arrow points at the data at early stage of adsorption film formation.

The dynamic surface elasticity and dynamic surface tension of dilute β-casein fibril dispersions were measured as a function of the number of purification cycles, concentration, solution ionic strength and surface age. The dynamic surface elasticity of an unpurified dispersion with a concentration of 0.01 mg/mL is a non-monotonic function of the surface age (Figure 3a). At the beginning of adsorption, it increases to 15 mN/m and decreases after that to approximately 12 mN/m. Such behavior is similar to that of native β-casein solutions at comparable protein concentrations. A local maximum of the dynamic surface elasticity was also observed for spread layers of this protein [48,49,50] and native β-casein solutions (Figure S4). This was explained by a transition from all trains’ conformation of the flexible protein in the surface layer to a thicker layer, with loops and tails in the distal region of the layer [39,48,51].

A close similarity of the dynamic surface elasticity of unpurified fibril dispersions and native protein solutions was previously observed for systems with β-lactoglobulin (BLG), lysozyme and bovine serum albumin (BSA), and is mainly caused by the influence of small peptides and unreacted proteins on the properties of fibril dispersions [31,32,33,34]. Although the dynamic surface properties of the solutions of native β- and κ-casein are similar, those of the unpurified fibril dispersions of these two proteins are not [35]. The dynamic surface elasticity of the dispersions of κ -casein fibrils changes monotonically with surface age and reaches values exceeding the results for the native protein solution [35]. The observed distinction can be caused by a lower propensity of β-casein to aggregation. As a result, the dispersions of β-casein fibrils contain higher concentrations of native protein molecules and their influence on the surface properties becomes stronger.

The local maximum of the dynamic surface elasticity is preserved even after one-time purification of the fibril dispersion, but the surface elasticity at the approach to equilibrium increases and reaches 25 mN/m (Figure 3a). Only two or more purifications lead to monotonic kinetic dependences of the surface elasticity and to higher values, as has been observed previously for dispersions of κ-casein fibrils [35]. At the same time, the changes in surface elasticity are slower for purified dispersions of β-casein fibrils. The highest surface elasticity of 48 mN/m is observed for twice purified fibril dispersions.

The surface tension of fibril dispersions also differs from the values for adsorption layers of native β-casein and reaches 56–58 mN/m (Figure 3b).

**Figure 4:**
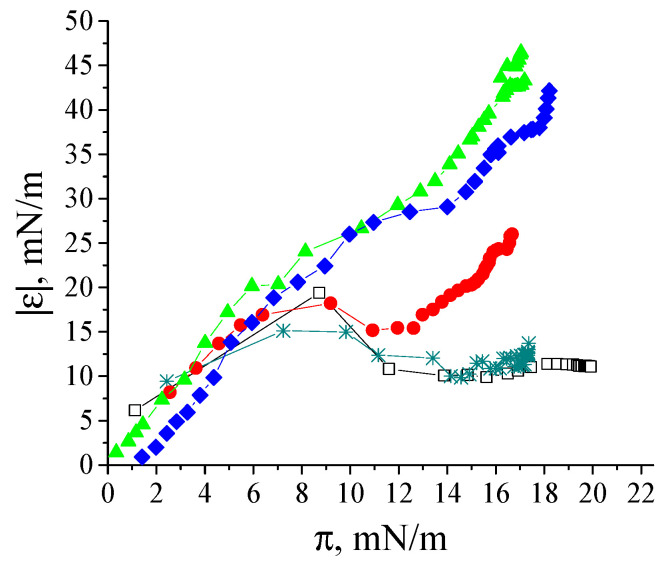
Dependence of dynamic surface elasticity on surface pressure of native β-casein solution (open black squares), unpurified fibril dispersion (cyan snowflakes), one-time (red circles), two-times (green triangles) and three-times (blue diamonds) purified fibril dispersions with a concentration of 0.01 mg/mL at pH 7.0 and with 0.1 M NaCl. The standard deviation of the measured parameters was lower than 5%.

In the case of a local equilibrium between the surface and subsurface layers, the plotting of the dependence of the dynamic surface elasticity on surface pressure allows elimination of the peculiarities of the adsorption kinetics and facilitates the comparison of different systems. The dependences of the dynamic surface elasticity on surface pressure can be divided into two parts (Figure 4). At π < 8 mN/m, the graphs corresponding to fibril dispersions and native β-casein solutions are rather close. Due to the small size of unreacted polypeptides, their adsorption proceeds more quickly and determines the surface properties at low surface ages and low surface pressures. The first step in the adsorption layer formation even for purified fibril dispersions can be connected with the adsorption of small polypeptides. On the other hand, at low surface concentrations, the primary structure of polypeptides can be more important than the organization of amino acid residues in the macromolecules. The main differences between the purified dispersions of β-casein fibrils and native protein solutions can be observed at π > 8 mN/m. It can be assumed that, as in the case of κ-casein aggregates [35], the increase in the dynamic surface elasticity in this range is connected with the fibril adsorption.

**Figure 5:**
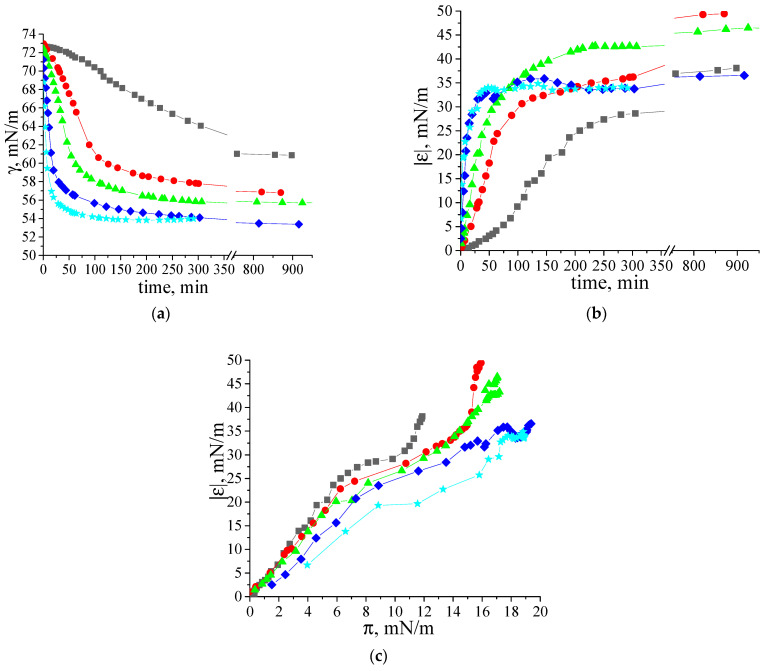
Kinetic dependences of (**a**) dynamic surface tension, (**b**) dynamic surface elasticity and (**c**) dependence of dynamic surface elasticity on surface pressure of two-times purified β-casein fibril dispersions with concentrations of 0.002 mg/mL (black squares), 0.005 mg/mL (red circles), 0.01 mg/mL (green triangles), 0.02 mg/mL (blue diamonds) and 0.05 mg/mL (cyan stars) at pH 7.0 and with 0.1 M NaCl. The standard deviation of the measured parameters was lower than 5%.

The effect of β-casein dispersion concentration on the dynamic surface properties is similar to that for κ-casein fibril dispersions [35]. The increase in concentration leads to more rapid changes in both the surface tension and dynamic surface elasticity (Figure 5a,b). For convenience, Figure 5a,b with a logarithmic time scale is presented in the (Supplementary Materials Figure S5). At the same time, the steady-state surface elasticity increases only up to a certain dispersion concentration, above which it starts to decrease (Figure 5b,c).

The highest values for dynamic surface elasticity (47–49 mN/m) correspond to adsorption layers of the dispersions with concentrations in the range of 0.005–0.01 mg/mL. This effect can be connected with the increase in the number of relatively thick regions in the adsorption layer (patches of multilayers), where the fibrils can take part in the mass exchange between different layers.

**Figure 6:**
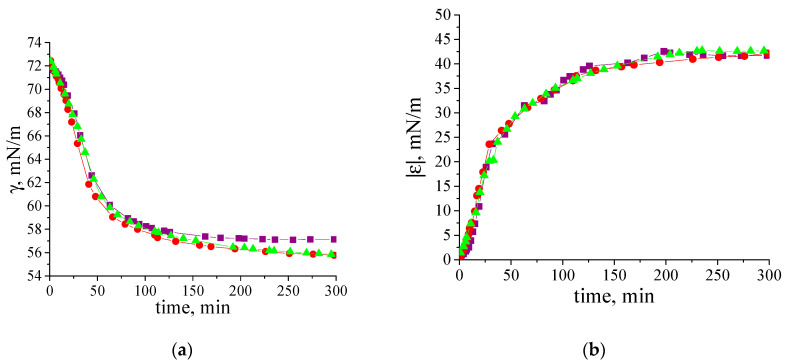
Kinetic dependences of (**a**) dynamic surface tension and (**b**) dynamic surface elasticity of two-times purified β-casein fibril dispersions (0.01 mg/mL) at pH 7.0 and with 0.01 M NaCl (purple squares), 0.05 M NaCl (red circles) and 0.1 M NaCl (green triangles). The standard deviation of the measured parameters was lower than 5%.

At pH 7, β-casein fibrils have a negative charge (the pI of β-casine is 4.8–5, Figure S6). However, a 10-fold increase in ionic strength does not significantly affect the kinetics of dynamic surface properties (Figure 6a,b). This behavior differs from some previously studied systems, such as κ-casein fibrils [33,34,35,52,53]. In the latter case, increasing ionic strength accelerates macromolecule absorption by reducing electrostatic adsorption barrier. The unexpected negligible effect of the adsorption barrier for the system under investigation can be explained by the special localization of charge within the larger adsorbing fibrils and their adsorption layer. Further investigation is required to understand this phenomenon. Note that a similar effect was also observed for the adsorption of BLG fibrils [34].

The AFM images confirm the adsorption of β-casein fibrils from the purified dispersions (Figure 2d,e). Note that images of the adsorption layers predominantly contain shorter fibrils (up to 400 nm) as compared to those in the bulk phase of the dispersion. A possible explanation is that only some parts of the fibrils can be visible, while other parts are hidden in the deeper regions of the adsorption layer under the influence of admixtures. The BAM images do not allow observation of the microscopic heterogeneity of the adsorption layer (Figure S7A,B).

**Figure 7:**
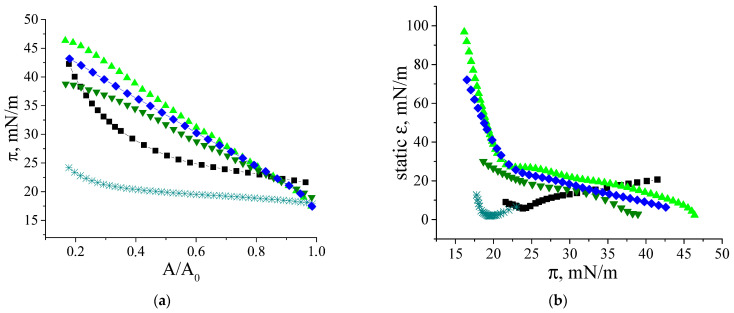
Compression isotherms (**a**) and dependences of static surface elasticity on surface pressure (**b**) of adsorption layers of native β-casein solution (black squares), unpurified fibril dispersion (cyan snowflakes), two-times purified fibril dispersion (green triangles up and green triangles down), three-times purified fibril dispersion (blue diamonds). Concentration of fibril dispersion was 0.01 mg/mL (black squares, cyan snowflakes, green triangles up, blue diamonds) and 0.05 mg/mL (green triangles down). pH and ionic strength were kept constant at 7.0 and 0.1 M, respectively. The accuracy of surface pressure measurements is ±0.2 mN/m.

The compression isotherms of the adsorption layers of β-casein molecules and fibrils give some additional information on the layer structure (Figure 7a). The difference in the behavior of native protein and fibrils is observed at the very beginning: the starting surface pressure for the native β-casein is 22 mN/m, whereas that for aggregate layers is lower—18 mN/m. The shape of the compression isotherms also depends on the type of particles forming the adsorption layer. For native protein solutions, the compression leads only to a moderate increase of the surface pressure until the surface area decreases two times. After that the growth of surface pressure accelerates and reaches values of approximately 44 mN/m at the end of compression. For unpurified dispersions, the surface pressure does not reach values higher than 25 mN/m even after compression down to 20% of the initial area (Figure 7a), indicating that the layer contains polypeptides of low molecular weight, which can partially desorb into the bulk after compression. In this case, the static surface elasticity is close to that of native protein layers over the entire range of investigated surface pressures (Figure 7b). The purified fibril dispersion demonstrates the third type of behavior. In this case, the surface pressure increases immediately after the start of compression and all the isotherms are almost linear. The two- and three-times purified fibril dispersions with a concentration of 0.01 mg/mL show a strong change in surface pressure, leading to much higher values at maximum compressions than for native β-casein adsorption layers. Up to a surface pressure of 33 mN/m, the static surface elasticity is much higher than for native β-casein films (Figure 7b). It can be assumed that, unlike the native protein layers, the adsorption layers of fibrils are almost incompressible. The layer compression results in a significant increase in the surface density and a more compact packing of the fibrils at the interface. Even a slight decrease in the surface area results in strong interactions between the aggregates. At further compression, the fibrils can form local multilayer regions. This tendency becomes more noticeable with the increase of fibril concentration and the compression isotherm for 0.05 mg/mL dispersion demonstrates a slower increase in surface pressure. Due to the displacement of fibrillar aggregates into the subsurface layer at high compressions, the static elasticity of the layers of two- and three-times purified dispersions does not exceed the values for native β-casein films. The formation of multilayers is supported by AFM data. The AFM images show that, after 5 times compression, the layer becomes non-uniform with some areas of local thickening, presumably corresponding to multilayers of fibrils (Figure 2f).

**Figure 8:**
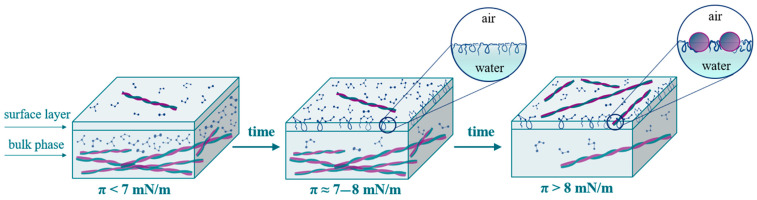
Scheme of the formation of adsorption layers of β-casein fibril dispersion.

The dynamic surface properties of β-casein fibril dispersions turn out to be significantly different from the properties of previously studied dispersions of globular protein fibrils. In the case of BLG and BSA purified fibril dispersions [33,34] the dynamic surface elasticity is close to the results for the corresponding native protein solutions. The interfering effect of surface-active impurities can be reduced only for spread fibril layers, where the concentration of polypeptide admixtures remains approximately equal to the value in the bulk solution. In this case, the surface elasticity of spread fibril layers turned out to be two times higher as compared to spread layers of native proteins. At the same time, the dynamic surface elasticity of adsorption layers is higher than that of native protein adsorption layers, as was observed for systems with lysozyme [34] and α-lactalbumin [54]. In this study, the same result was obtained for the layers of β-casein and its fibrils. While, in the case of spread and adsorption layers of lysozyme and α-lactalbumin fibrils, the differences in the surface elasticity with the results for native protein solutions were only 10–40%, the dynamic surface elasticity for the layers of β-casein aggregates is approximately four times higher than that of native protein layers. The results obtained show that, at the beginning of adsorption, the surface layer contains mainly peptides of low molecular weight and unreacted protein molecules. Some flexible parts of these molecules can be displaced from the proximal region of the surface layer into the distal one as loops and tails, leading to a local maximum for surface elasticity (Figure 8). The adsorption of fibrils probably occurs mainly with a short delay when the surface pressure exceeds approximately 7 mN/m.

## 4. Conclusions

The dynamic surface properties of aqueous β-casein fibril dispersions depend strongly on the purity of the dispersion, the total protein concentration and solution ionic strength. Some similarities were discovered with the surface properties of fibril dispersions of another intrinsically disordered protein—κ-casein. At the same time, the dynamic surface properties of the fibril dispersions of globular proteins were remarkably different. The main distinctions in the aggregate size and the kinetic dependencies of the dynamic surface elasticity between the fibril dispersions of β- and κ-caseins are due to the differences in the protein aggregation propensity and a relatively high content of unreacted molecules in the case of β-casein dispersions. As in the case of κ-casein fibril dispersions, the surface properties of purified β-casein fibril dispersions differ significantly from the properties of native protein solutions. The dynamic surface elasticity values of the purified fibril dispersions were twice as high as those for the native protein solutions in the region of the local maximum of the elasticity and four times higher at the approach to equilibrium. Such a strong relative increase in the surface elasticity after fibril formation has not been observed earlier for any other aqueous protein system.

## Data Availability

The data presented in this study are openly available in the article.

## Supplementary Materials

The following supporting information can be downloaded at: https://www.mdpi.com/article/10.3390/polym17081075/s1, Table S1. Protein concentrations before and after purification; Figure S1: Fluorescence spectra of ThT after interaction with dispersions of β-casein fibrils of different purity; Figure S2: HPLC chromatogram of the native β-casein (black line), unpurified dispersion after synthesis (red line) and two-times purified dispersion (green line); Figure S3: (A) Fluorescence spectra and (B) excitation spectra of ThT after interaction with β-casein at different incubation times at pH 2 and 90 °C; Figure S4: Dependence of dynamic surface elasticity on surface pressure of native β-casein solutions with concentrations of 0.001 mg/mL (half open black squares), 0.002 mg/mL (half open red circles), 0.01 mg/mL (half open green triangles), 0.015 mg/mL (half open blue diamonds) and 0.04 mg/mL (half open cyan asterisks) at pH 7.0; Figure S5. Kinetic dependences of (a) dynamic surface tension and (b) dynamic surface elasticity of two-times purified β-casein fibril dispersions with concentrations of 0.002 mg/mL (black squares), 0.005 mg/mL (red circles), 0.01 mg/mL (green triangles), 0.02 mg/mL (blue diamonds) and 0.05 mg/mL (cyan stars) at pH 7.0 and with 0.1 M NaCl. Figure S6: Dependence of zeta potential of two-times purified β-casein fibrils on concentration of NaCl; Figure S7: BAM images of (A) adsorption layer of unpurified dispersion of β-casein fibrils; (B) two-times purified dispersion of β-casein fibrils.
