# Supplementary material for: Dynamic Properties of β-Casein Fibril Adsorption Layers at the Air–Water Interface
Source: Polymers (Basel). 2025 Apr 16;17(8):1075. doi: 10.3390/polym17081075 (PMC12030679; doi:10.3390/polym17081075)
Supplement: Supplementary file 1 [file polymers-17-01075-s001.zip › polymers-3528768-supplementary.pdf]

Supplementary Materials

Dynamic Properties of β-Casein Fibril Adsorption Layers at the Air–Water Interface

Anastasiya R. Rafikova [1], Olga Y. Milyaeva [1,*], Giuseppe Loglio [2], Reinhard Miller [3], Zhili Wan [4] and Boris A. Noskov [1]

[1] Department of Colloid Chemistry, St. Petersburg State University, Universitetsky pr. 26, 198504 Saint Petersburg, Russia; nastya.rafikova.2000@mail.ru (A.R.R.); b.noskov@spbu.ru (B.A.N.)
[2] Institute of Condensed Matter Chemistry and Technologies for Energy, 16149 Genoa, Italy; giuseppe.loglio@ge.icmate.cnr.it
[3] Institute of Condensed Matter Physics, Technische Universität Darmstadt, D-64289 Darmstadt, Germany; reinhard.miller@pkm.tu-darmstadt.de
[4] School of Food Science and Engineering, South China University of Technology, Guangzhou 510640, China; zhiliwan@scut.edu.cn
* Correspondence: o.milyaeva@spbu.ru; Tel.: +7-906-249-12-97

Composition and purity of the samples

The fibril dispersions were obtained from concentrated β-casein (Sigma-Aldrich, Germany, purity >98%) solutions. The total protein concentration in dispersions and solutions was determined gravimetrically (Table S1).

Table S1. Protein concentrations before and after purification.

№	Sample description	Concentration, mg/ml
1	Unpurified dispersion after synthesis	9.4±0.05
2	The upper fraction after the first centrifugation	7.7±0.05
3	One-time purified dispersion	1.7±0.05
4	The upper fraction after the second centrifugation	0.5±0.05
5	Two-time purified dispersion	1.2±0.05

With the aim to evaluate the fibril concentration in the samples they were characterized by thioflavin T (ThT) fluorescence assay. Before the measurements the concentration of all samples was reduced to 1.7 mg/ml. The TNT Concentration was $1*10^{-5}$ M.

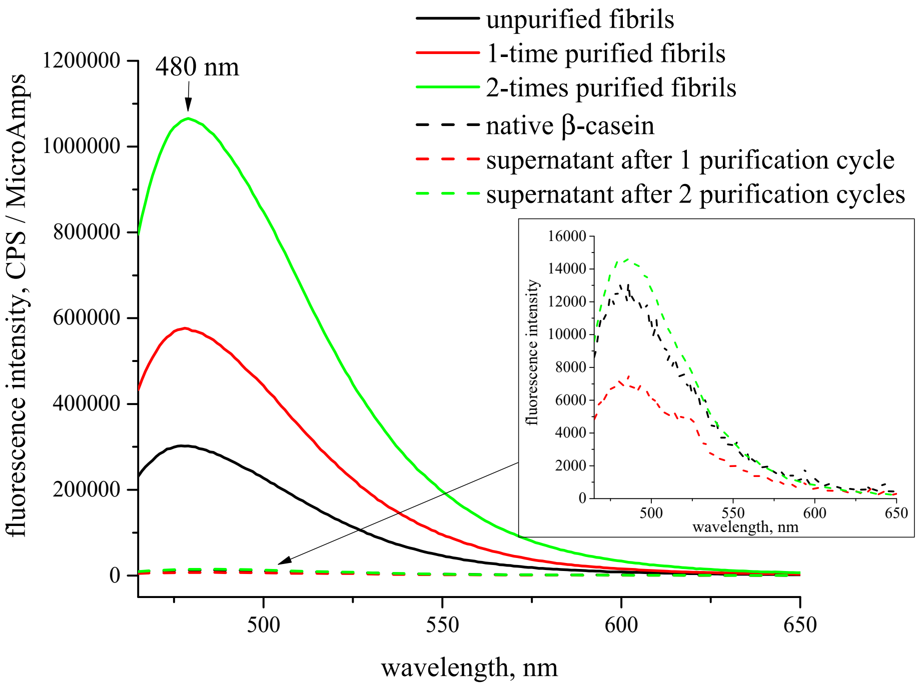

Figure S1. Fluorescence spectra of ThT after interaction with dispersions of β-casein fibrils of different purity.

ThT molecules in aqueous solutions exhibit weak fluorescence with a quantum yield of about 10^{-4}. The maximum absorption of aqueous solutions of the free dye is observed at 412 nm (not shown), and the maximum in the emission spectrum corresponds to 480 nm. The ThT interaction with the extended β-sheet structure of fibrils leads to an increase of the ThT fluorescence intensity by several orders of the magnitude at 480 nm, and the absorption band maximum undergoes a shift to the long–wavelength region 445-450 nm. One can observe this effect for initial unpurified dispersions of fibrils, for the dispersions after one and two

centrifugation cycles (Figure S1). For the upper supernatant fractions after the first and second centrifugation cycles the ThT fluorescence intensity is 2 orders of magnitude lower and close to the values for the mixtures of ThT with the native protein (Figure S1). The weak fluorescence is caused by a certain number of β-sheets as regular elements of the secondary structure.

The heating in acidic conditions for 48 hours leads to the conversion of less than 20 % of the initial protein to fibrils. From 82% (after first purification cycle) to 87 % (after first and second purification cycle) of the substance in dispersion after the synthesis, as shown by the fluorescence spectra, have a non-fibrillar structure.

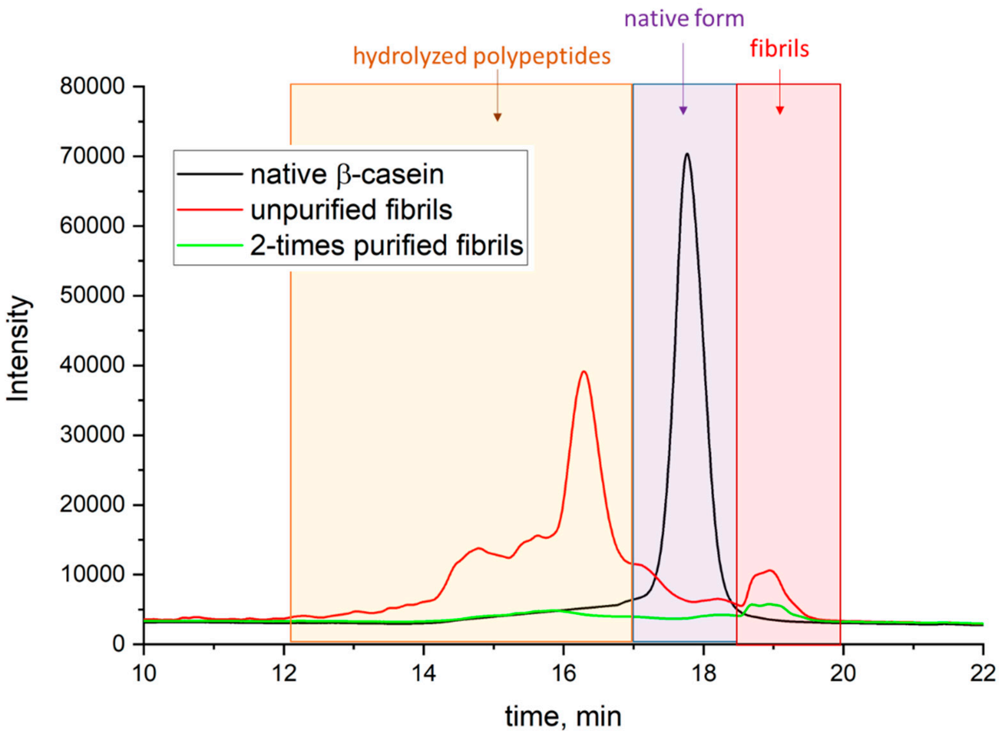

Figure S2. HPLC chromatogram of the native β-casein (black line), unpurified dispersion after synthesis (red line) and two-times purified dispersion (green line).

The High-Performance Liquid Chromatography (HPLC) was performed for a preliminary evaluation of the sample composition. The samples were run on a Shimadzu LC-20 system through Phenomenex Columns HPLC (150 × 2.0 mm) and were detected at 280 nm using a uvv104m detector (Akvilon, Russia). The total run time for the samples was 40 min. Two solvent systems were used for the run. The first solvent (Solvent A) was acetonitrile/H_2O/trifluoroacetic acid in a ratio of 10/90/0.05 v/v, the second one (Solvent B) - acetonitrile/H_2O in a ratio of 90/10 v/v.

Distinct peaks within each HPLC trace (Figure S2) were observed showing the change in the sample composition before and after the synthesis and purification. The chromatogram for the native protein in Figure 2S shows one sharp peak at ~17.5 min. After the thermal treatment this peak disappears and one can see wide peaks eluted between 12 and 16.5 min and one peak at 19 min. The wide peaks can be attributed to a mixture of polypeptides of different molecular weight. The last peak indicates the formation of fibrils - the aggregates with a size bigger than the initial protein. After 2 cycles of purification by centrifugation only this peak at 19 min is noticeable

in the chromatogram. These findings prove the effectiveness of the applied purification procedure.

Characterization of the rate of fibril formation

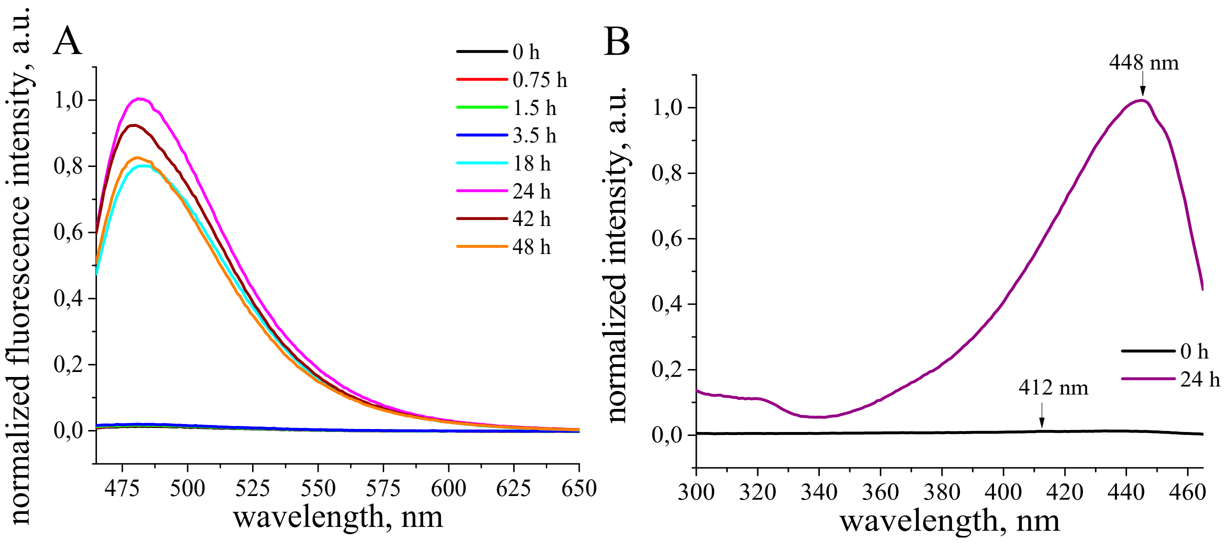

Figure S3. A) Fluorescence spectra and B) excitation spectra of ThT after interaction with β-casein at different incubation times at pH 2 and 90°C.

Dynamic surface properties of native protein

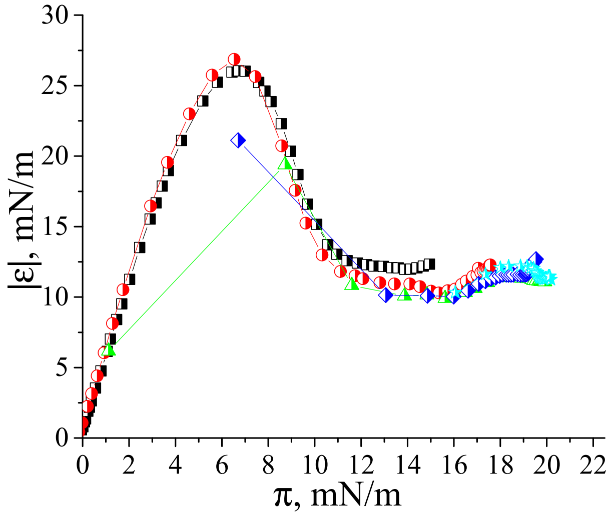

Figure S4. Dependence of dynamic surface elasticity on surface pressure of native β-casein solutions with concentrations of 0.001 mg/mL (half open black squares), 0.002 mg/mL (half open red circles), 0.01 mg/mL (half open green triangles), 0.015 mg/mL (half open blue diamonds) and 0.04 mg/mL (half open cyan asterisks) at pH 7.0.

Properties of the fibrils

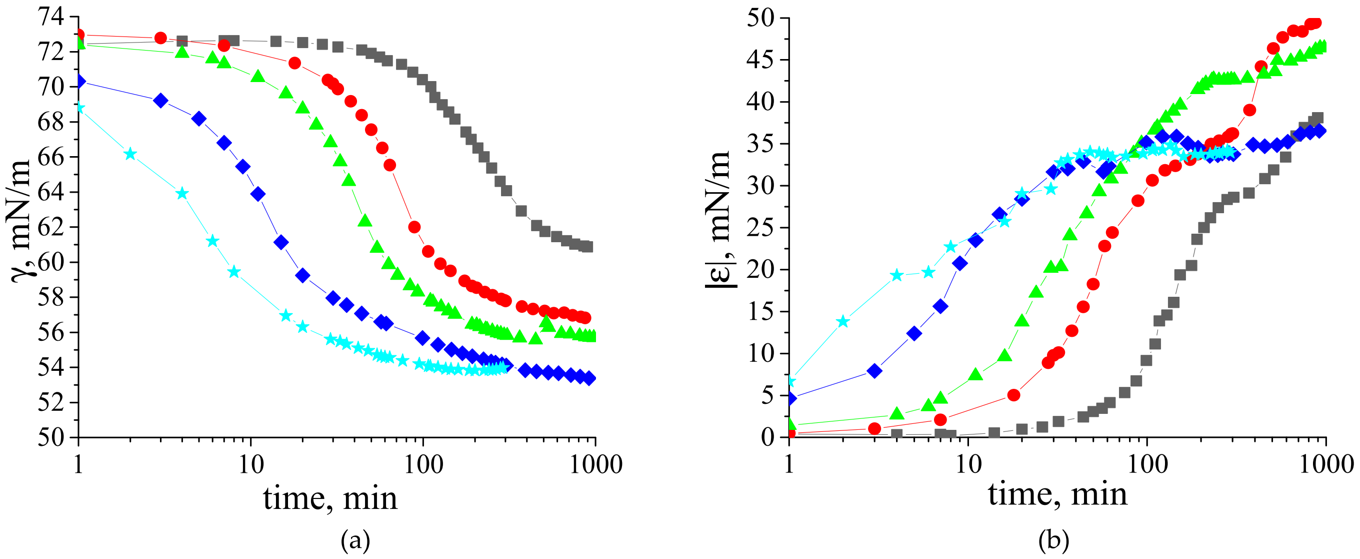

Figure S5. Kinetic dependences of (a) dynamic surface tension and (b) dynamic surface elasticity of two-times purified β-casein fibril dispersions with concentrations of 0.002 mg/mL (black squares), 0.005 mg/mL (red circles), 0.01 mg/mL (green triangles), 0.02 mg/mL (blue diamonds) and 0.05 mg/mL (cyan stars) at pH 7.0 and with 0.1 M NaCl.

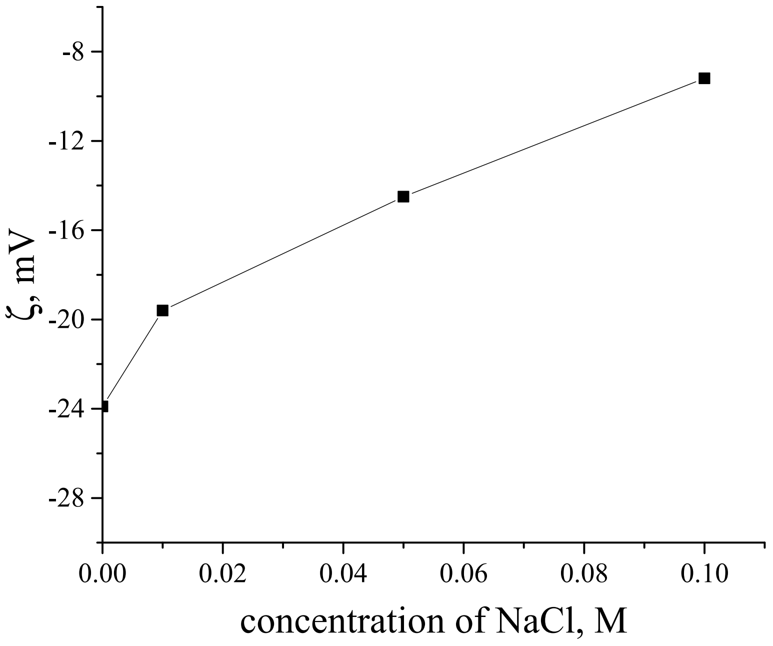

Figure S6. Dependence of zeta potential of two-times purified β-casein fibrils on NaCl concentration.

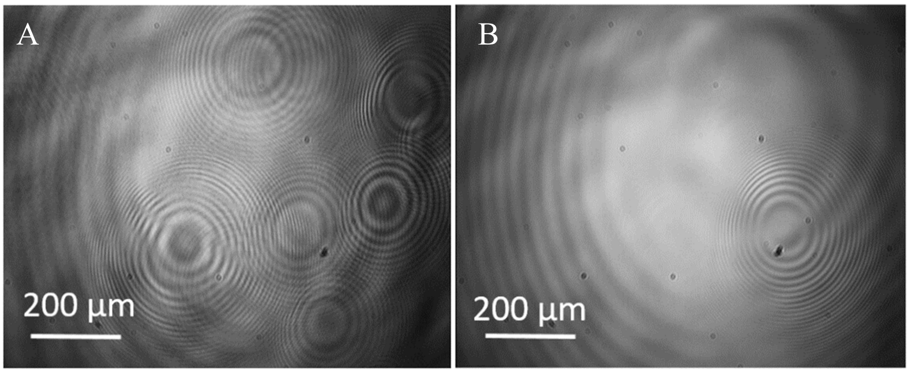

Figure S7. BAM images of A) adsorption layer of unpurified dispersion of β-casein fibrils; B) two-times purified dispersion of β-casein fibrils.
